# Coexistence of Ovarian Endometrioma and Ovarian Pregnancy: A Case Report

**DOI:** 10.7759/cureus.35608

**Published:** 2023-02-28

**Authors:** Yoko Aoyagi, Kentaro Kai, Saki Aso, Masakazu Nishida, Yasushi Kawano

**Affiliations:** 1 Department of Obstetrics and Gynecology, Oita University Faculty of Medicine, Yufu, JPN

**Keywords:** case report, endometriosis, ovarian pregnancy, hemoperitoneum, laparoscopic treatment, ovarian endometrioma

## Abstract

Both ovarian pregnancy and endometrioma can rupture and cause life-threatening hemoperitoneum. However, little is known about their coexistence. We report the case of a 34-year-old Japanese woman with a life-threatening hemoperitoneum in the first trimester coexisting with ovarian endometrioma and ovarian pregnancy. The patient was hospitalized in our department for acute hypogastric pain and massive hemoperitoneum during pregnancy. She had a history of miscarriage at eight weeks of gestation one year prior. Her serum beta-human chorionic gonadotropin (hCG) level was >2,000 mIU/mL. Also, a transvaginal ultrasound showed an empty uterus, an intact right ovary, an inhomogeneous left ovary, and a massive hemoperitoneum. An exploratory laparoscopy revealed a rupture of the left ovarian endometrioma, a left corpus luteal cyst, and intraperitoneal bleeding of approximately 1,200 mL. However, no ectopic lesions were observed. Microscopic examination revealed an endometriotic cyst with decidual changes in the stroma, a corpus luteal cyst, and chorionic villi with hemorrhage. Serum beta-hCG levels became negative on the 27th postoperative day. The postoperative course was uneventful. This case shows that, in addition to the differential diagnosis of ovarian pregnancy from ovarian endometrioma, clinicians should consider the coexistence of both conditions.

## Introduction

Ectopic pregnancy with hemoperitoneum is the leading cause of maternal mortality in the first trimester [1]. Although ovarian pregnancy is a rare variant of ectopic pregnancy, its incidence has increased in recent years [2]. It often clinically manifests as a life-threatening hemoperitoneum because of the diagnostic delay and the ruptured ectopic lesion [3]. Preoperative diagnosis of ovarian pregnancy is challenging because it morphologically and sonographically mimics a corpus luteum cyst, ovarian tumor, and intact or ruptured tubal pregnancy [4].

Further, ovarian endometrioma is a common subtype of endometriosis. It is a benign, multicyclic tumor arising from ectopic endometrial tissue within the ovary [5]. Most ovarian endometriomas are detected by transvaginal ultrasonography, but laparoscopic visualization and histological verification remain the gold standard for diagnosis. Ovarian endometrioma can cause hemoperitoneum due to rupture with an estimated incidence of less than 3% among women with ovarian endometrioma [6]. However, no specific recommendation is available for asymptomatic ovarian endometrioma during pregnancy [7].

Here, we report the case of a woman with a life-threatening hemoperitoneum in the first trimester coexisting with ovarian endometrioma and ovarian pregnancy.

## Case presentation

**Figure 1:**
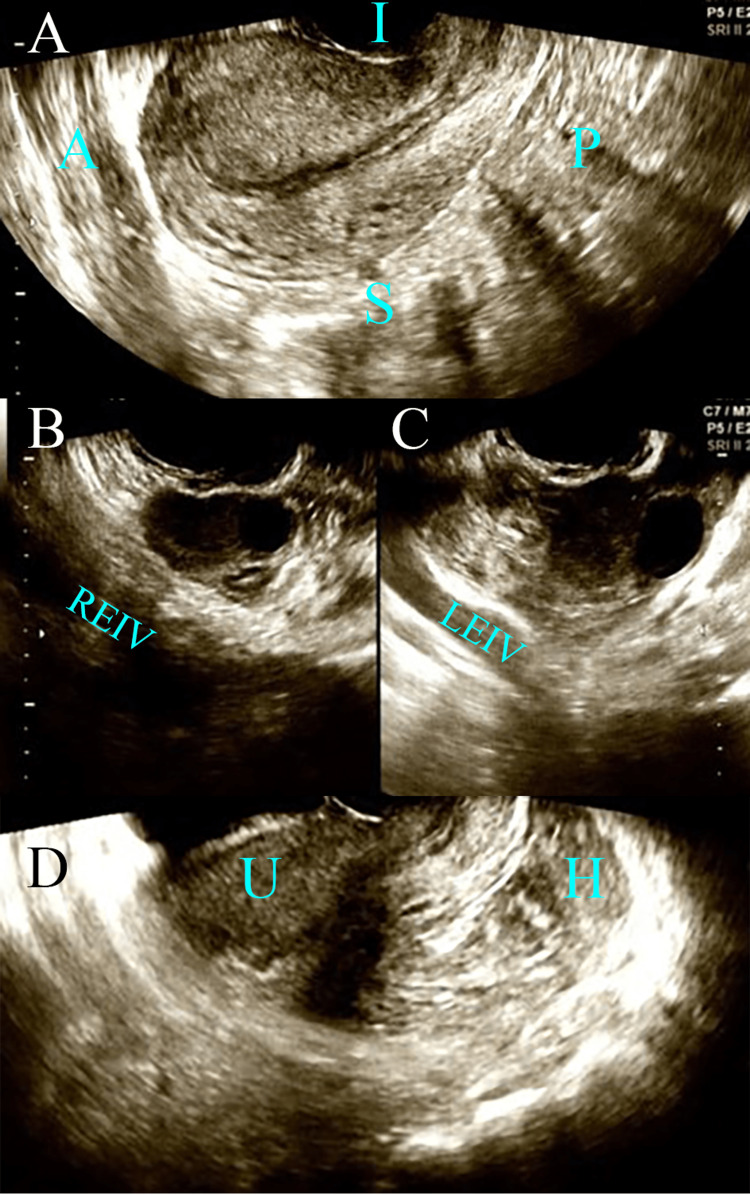
Preoperative images. (A) Transvaginal ultrasound shows thin endometrial lining, indicating no evidence of an intrauterine pregnancy. (B) Normal findings in the right ovary. (C) An inhomogeneous extra-ovarian mass was seen in the left ovary. (D) A hemoperitoneum (free fluid) newly appeared in her cul-de-sac. A, anterior; P, posterior; I, inferior; S, superior; REIV, right external iliac vein; LEIV, left external iliac vein; U, uterus; H, hemoperitoneum.

A 34-year-old Japanese woman (gravida 2, para 0) was hospitalized in our department for acute hypogastric pain and massive hemoperitoneum during pregnancy. She had conceived by the first trial of human menopausal gonadotropin (hMG)-human chorionic gonadotropin (hCG) therapy for infertility and was five weeks and six days of gestational age based on the date of the last period. She had visited a primary care doctor two days prior to presentation because of a positive pregnancy test result. Transvaginal ultrasound revealed the following: a thin endometrial lining without a gestational sac in the uterine cavity (Figure 1A), normal findings in the right ovary (Figure 1B), and an inhomogeneous extra-ovarian mass in the left ovary (Figure 1C). She was advised to come for a follow-up visit the following week. However, the following day, she presented with acute hypogastric pain and then visited a primary care doctor. A massive new hemoperitoneum appeared in her cul-de-sac (Figure 1D). She had a history of miscarriage at eight weeks of gestation one year before.

In the emergency room, she was in agony and appeared pale with abdominal distention. Her vital signs were as follows: blood pressure, 97/58 mmHg; pulse, 73 beats/min; respiratory rate, 14 breaths/min; body temperature, 36.4°C; and SpO2 (pulse oximetry), 100% (room air). Transvaginal ultrasound findings were similar to those of the primary care doctor. Laboratory tests showed the following: white cell count, 27,170 per mm3; hemoglobin, 9.0 g/dL; hematocrit, 27.0%; platelet count, 210,000 per mm3; C-reactive protein, 0.09 mg/dL; procalcitonin, <0.02 ng/mL; and beta-hCG, 2,803 mIU/mL.

**Figure 2:**
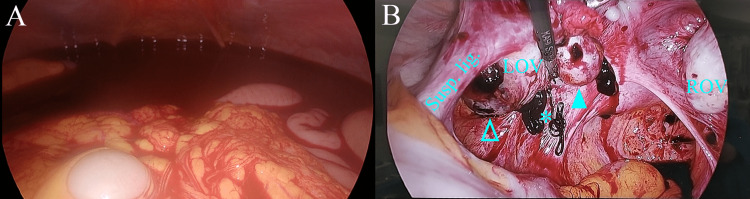
Intraoperative findings (A) Hemoperitoneum is noted during laparoscopy. (B) Overview of the pelvis after suctioning of pooled blood (~1,200 mL). Two distinct ovarian masses are noted (arrowheads): a partly dark-red colored ovarian mass (open arrowhead) is located on the suspensory ligament side of the left ovary with leakage of chocolate-like intratumor fluid (*), and a well-defined smooth-surfaced mass without rupture is located on the ovarian ligament side of the left ovary (closed arrowhead).

Due to the absence of evidence of intrauterine pregnancy and the presence of an inhomogeneous extra-ovarian adnexal mass and intraperitoneal bleeding, we performed exploratory laparoscopy. Laparoscopic findings revealed a large hemoperitoneum (approximately 1,200 mL) (Figure 2A). After suctioning pooled blood from the cul-de-sac, two distinct masses were noticed in her left ovary (Figure 2B). One was suspected to be an endometriotic cyst (open arrowhead) with rupture (asterisk), and the other was a smooth-surfaced mass without rupture (closed arrowhead). We first enucleated the smooth-surfaced mass, but neither a gestational sac nor villi were noted, indicating a corpus luteal cyst. Next, we enucleated the ovarian endometrioma, which includes blood clots on its surface and in the cyst. Despite a careful observation of the pelvic and abdominal cavities, an ectopic pregnancy site was not identified. The tentative intraoperative diagnosis was a corpus luteal cyst, a rupture of the ovarian endometrioma, and an ectopic pregnancy at an unknown site. The duration of surgery was two hours and 27 minutes, and intraoperative blood loss was minimal and uncountable. However, the patient’s beta-hCG levels decreased immediately and became negative on postoperative Day 27. Her postoperative course was uneventful.

**Figure 3:**
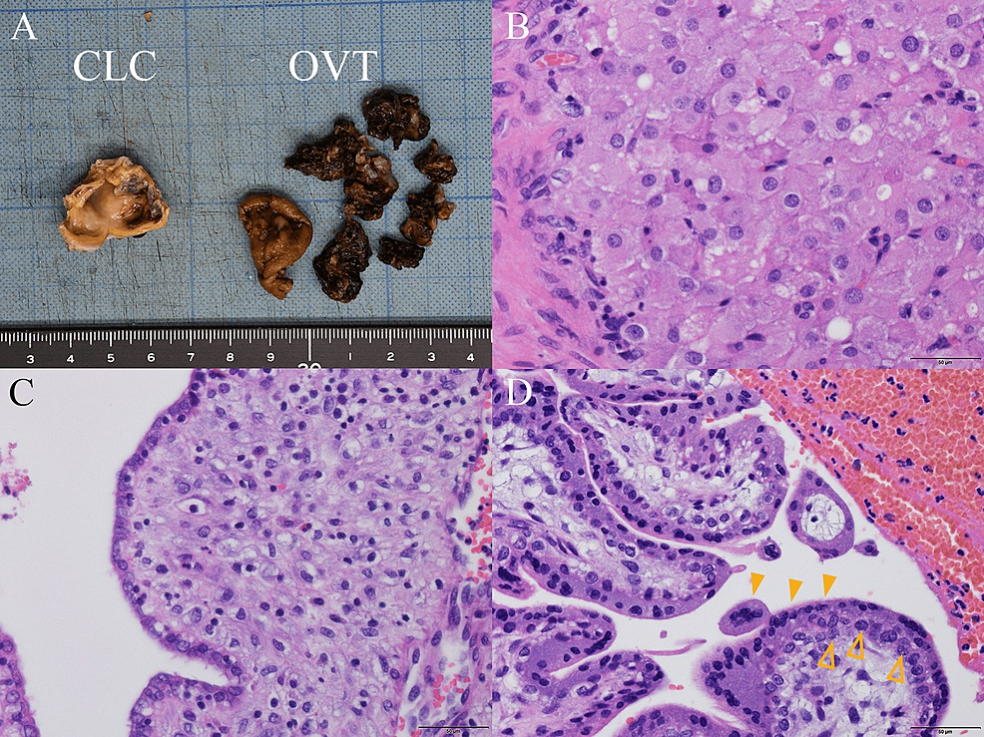
Pathological findings (A) Macroscopic view of resected specimens. The cut surface of the corpus luteal cyst (CLC) is yellow and convoluted with a central cystic and hemorrhagic cavity. The lumen of enucleated ovarian tumor (OVT) includes a blood clot, imparting the macroscopic appearance of a chocolate cyst. Ruler: cm/mm. (B) The microscopic appearance of CLC shows large, luteinized granulosa cells with small uniform nuclei and abundant large eosinophilic cytoplasm. (C) The microscopic appearance of OVT reveals a single layer of columnar epithelium and stromal cells with prominent decidual change (large rounded stromal cells with abundant eosinophilic cytoplasm, large round nuclei, and nucleoli), indicating ovarian endometrioma. (D) Some chorionic villi are observed within the hemorrhage on the same ovary, indicating ovarian pregnancy (open arrowheads: cytotrophoblasts; closed arrowheads: syncytiotrophoblasts). Scale bar: 50 µm.

The macroscopic view is shown in Figure 3A. Microscopic examination with hematoxylin and eosin staining revealed a corpus luteal cyst (Figure 3B) and an endometriotic cyst with decidual changes (Figure 3C). Furthermore, chorionic villi were detected in the disrupted endometriotic cyst specimen surrounded by hemorrhage (Figure 3D).

Owing to the retrospective nature of this case report, the need for approval by the institutional review board was waived. Written informed consent was obtained from the patient for their anonymized information to be published in this article.

## Discussion

This report describes a case of massive hemoperitoneum in the first trimester, secondary to a ruptured ovarian endometrioma coexisting with ovarian pregnancy. We performed a laparoscopic ovarian cystectomy for hemostasis and endometrioma treatment but did not find an ectopic lesion intraoperatively. However, a microscopic examination of the resected specimen revealed an ectopic endometrial epithelium, decidualized stroma, and chorionic villi. To our knowledge, this is the first report of the coexistence of a ruptured ovarian endometrioma and an unruptured ovarian pregnancy.

We conducted a literature search and review regarding the coexistence of ovarian pregnancy and ovarian endometrioma in PubMed/MEDLINE and Google Scholar, and the results are summarized in Table 1. We used the terms “endometriosis” and “ovarian pregnancy” to search for literature in English, with abstracts available and without publication date filters. All patients manifested hemoperitoneum with abdominal pain, except those in Punnonen and Laurén’s report; they examined 41 women with ovarian pregnancy, but individual patient information was not provided [8]. The surgical approach involved a less invasive transition: laparotomic resection of internal genitals [9], laparotomic adnexectomy [8,10], and laparoscopic ovarian sparing surgery. Coexistence with ovarian endometrioma has been reported to be an incidental finding of ovarian pregnancy. However, in this case, ruptured ovarian endometrioma was the main pathology.

**Table 1:** Histologically confirmed cases of ovarian pregnancy coexisting with ovarian endometrioma on the same ovary y, year; GA, gestational age; wk, weeks; NA, not applicable; BSO, bilateral salpingo-oophorectomy; U, unilateral; Lt, left; Rt, right; LSO, left salpingo-oophorectomy.

Reference	Year	Episode	Age (y)	GA (wk)	Clinical presentations	Intraoperative findings	Surgical interventions	Pathological findings
Durburg et al. [9]	1958		32	15	Paraumbilical pain, weakness, and syncope	Hemoperitoneum, fibroids, undetectable rt. ovary, and hemorrhagic mass in a cul-de-sac	Laparotomic supracervical hysterectomy +BSO	Rt. ovarian pregnancy (ruptured) and endometriosis with the decidual change of both ovaries and lt. tube
Punnonen and Laurén [8]	1982	I	NA	NA	NA	NA	Laparotomic USO	Coexisting foci of endometriosis
		II	NA	NA	NA	NA	Laparotomic USO	Coexisting foci of endometriosis
Toki et al. [10]	1998		29	7	Lower lt. quadrant dull pain, genital bleeding, and amenorrhea	Ruptured lt. ovarian mass and hemoperitoneum (~200 mL)	Laparotomic LSO	Lt. ovarian pregnancy (ruptured) and endometriosis with decidual change
This case	2023		34	5	Hypogastric pain	Ruptured lt. endometrioma, hemoperitoneum (~1200 mL), and undetectable ectopic lesion	Laparoscopic lt. ovarian cystectomy	Lt. ovarian pregnancy, normal corpus luteum cyst, and endometriosis with decidual change (ruptured)

The ovary is the most common anatomic site of endometriosis, which is laparoscopically diagnosed. Ovarian endometrioma accounts for approximately 67% of all endometriosis sites [11]. Sampson’s theory is widely accepted among the different theories on the pathogenesis of endometriosis. It states that endometriosis is caused by ectopic implantation of retrograde menstrual blood [12]. However, the pathogenesis of ovarian pregnancy is divided into primary and secondary types. In the primary type, interfollicular fertilization occurs and the fertilized ovum develops in situ. In the secondary type, extrafollicular fertilization occurs, usually in a tube, with a fertilized ovum implanting on the ovary. A meta-analysis of 15 studies exploring the association between endometriosis and ectopic pregnancy showed that endometriosis is associated with an increased risk of ectopic pregnancy, with an odds ratio of 2.66 (95% confidence interval = 1.14-6.21) [13]. However, the site-specific association between ovarian endometrioma and pregnancy remains unknown.

The definitive diagnosis of ovarian pregnancy is based on intraoperative-anatomic and postoperative-histologic findings. The four classical diagnostic criteria proposed by Spielberg are as follows: (i) the fallopian tube should be intact and separate from the ovary, (ii) the gestational sac should occupy the normal position in the ovarian pelvis, (iii) the gestation should be connected to the uterus by the ovarian ligament, and (iv) ovarian tissue must be present in the specimen connected to the gestational sac [14]. However, these criteria are inappropriate because of the advancement and popularization of minimally invasive approaches in surgery and ovarian hyperstimulation in assisted reproductive technologies [4]. Bontis et al. proposed the modified Spielberg criteria to satisfy intrafollicular ovarian pregnancy in cases of hyperovulated ovaries [15]. Chelmow et al. later proposed a modification of Spielberg’s fourth criterion to satisfy the laparoscopic approach as follows: laparoscopically directed biopsy of the affected area demonstrated chorionic villi within the ovarian lesion [16]. Additionally, some researchers have reported a case of ovarian pregnancy coexisting with ovarian dermoid (mature cystic teratoma) [17]. We further propose modifying Spielberg’s fourth criterion to include “coexisting with other tumor entities,” not only “within the ovarian lesion.”

## Conclusions

We report the case of a woman with a life-threatening hemoperitoneum caused by a ruptured ovarian endometrioma coexisting with an unruptured ovarian pregnancy. Although well-trained surgeons are needed, exploratory laparoscopy is a useful therapeutic approach when a pre- and intra-operative diagnosis of ovarian pregnancy is difficult due to uncommon and complicated intrapelvic findings. Given the increased prevalence of the two diseases in this case, clinicians should pay attention to the differential diagnosis of ovarian pregnancy from ovarian endometrioma and the coexistence of these two diseases.
